# Long-term effects on PTH and mineral metabolism of 1.25 versus 1.75 mmol/L dialysate calcium in peritoneal dialysis patients: a meta-analysis

**DOI:** 10.1186/s12882-019-1388-9

**Published:** 2019-06-11

**Authors:** Liqin Jin, Jingjing Zhou, Feng Shao, Fan Yang

**Affiliations:** Nephrology Center, Beijing Luhe Hospital Capital Medical University, Beijing, 101149 China

**Keywords:** Dialysate calcium, Intact parathyroid hormone, Meta-analysis, Peritoneal dialysis

## Abstract

**Background:**

This study aimed to compare 1.25 and 1.75 mmol/L dialysate calcium for their effects on parathyroid hormone (PTH) and mineral metabolism in peritoneal dialysis (PD).

**Methods:**

The PubMed, Cochrane Library, and EmBase databases were searched from inception to October 2016. Methodological quality assessment of the included studies was performed using the risk of bias tool of the Review Manager software. The meta-analysis was carried out with the Stata12.0 software. Subgroup analysis was performed by study design [randomized controlled trial (RCT) and non-RCT]. Odds ratios or standardized mean differences were used to assess the outcome measures, including intact parathyroid hormone (i-PTH) levels, serum total calcium amounts, ionized calcium levels, phosphate concentrations, and peritonitis episodes.

**Results:**

Seven studies were enrolled in the synthesized analysis, including 4 RCTs and 3 non-RCTs. All studies compared 1.25 mmol/L and 1.75 mmol/L dialysate calcium for PD. Pooled analysis revealed that 1.75 mmol/L dialysate calcium significantly reduced i-PTH levels compared with the 1.25 mmol/L dose in PD patients. However, 1.25 mmol/L dialysate calcium was superior to the 1.75 mmol/L dose in decreasing the levels of serum total calcium and ionized calcium in PD patients. No significant differences in phosphate amounts and peritonitis episodes were observed between the two groups.

**Conclusion:**

These findings indicated that 1.75 mmol/L dialysate calcium is more appropriate for PD patients with secondary hyperparathyroidism. Meanwhile, 1.25 mmol/L dialysate calcium is more favorable to PD patients with secondary hypercalcemia. However, further well-designed and high-quality studies are required to validate these findings.

## Background

Peritoneal dialysis (PD) is an effective therapeutic method for azotemia induced by end stage renal failure (ESRF). However, PD is often accompanied by calcium–phosphorus and parathyroid hormone metabolism disorders [1, 2]. These ailments lead to hypocalcemia and secondary hyperparathyroidism, which in turn can become tertiary and cause hypercalcemia [3, 4]. Meanwhile, excessive calcium amounts are associated with risk of renal osteodystrophy [5, 6], adynamic bone disease [7], and metastatic calcification [6]. Furthermore, severe calcium–phosphorus metabolism impairment may induce unacceptably high cardiovascular morbidity and mortality [4, 8, 9].

Calcium concentration in the dialysate is a pivotal factor influencing serum calcium, phosphate, and parathyroid hormone (PTH) levels. Calcium levels in the dialysate vary and include l.0 mmol/L, 1.25 mmol/l, 1.5 mmol/L, and 1.75 mmol/L, with 1.25 and 1.75 mmol/L most widely used in commercially available PD solutions. Generally speaking, 1.75 mmol/L dialysate calcium, which is considered the standard dialysate calcium in many counties, may produce soft-tissue calcification and adynamic bone disease; meanwhile, 1.25 mmol/L dialysate calcium may cause hyperparathyroidism and acute arrhythmias.

A few trials have compared 1.75 mmol/L and 1.25 mmol/L dialysate calcium levels for the treatment of patients with ESRF, assessing their effects on health indexes such as serum calcium and intact parathyroid hormone (i-PTH). However, the optimal concentration remains unclear.

Only one meta-analysis of low versus standard dialysate calcium in PD was reported [10]. This study found that low dialysate calcium was superior to the standard dose in decreasing serum total calcium levels in PD patients, while the effects on i-PTH levels and peritonitis episodes remain controversial. Of these, i-PTH is an important factor in assessing treatment safety and identifying the required calcium concentration; peritonitis is the most common complication occurring during PD. Several studies [11–13] assessed the effects of different dialysate calcium concentrations in PD patients and reported inconsistent results. Therefore, it was necessary to perform an updated meta-analysis to evaluate the optimal dialysate calcium concentration for PD patients.

## Methods

This meta-analysis was performed in accordance with the Preferred Reporting Items for Systematic Reviews and Meta-Analyses statement [14].

### Literature search strategy

The following databases were electronically searched from inception to October 2016: PubMed, EmBase, and Cochrane Central Register of Controlled Trials. All studies comparing 1.25 mmol/L with 1.75 mmol/L dialysate calcium for PD were searched in the above electronic databases by two authors independently. MeSH/Entree and free word retrievals were combined to search the literature as much as possible. The search terms were as follows: “Peritoneal Dialysis” AND “calcium dialysate” AND (“l.0 mmol/l” OR “1.25 mmol/l” OR “1.5 mmol/l” OR “1.75 mmol/l”). To identify additional reports, the reference lists of all retrieved studies and published reviews/meta-analyses were manually searched, and all identified relevant articles were included.

### Eligibility criteria

The inclusion criteria for the current study were: (1) participants administered PD or continuous ambulatory peritoneal dialysis (CAPD); (2) 1.25 mmol/L and 1.75 mmol/L dialysate calcium respectively used in the two groups; (3) follow-up exceeding 12 months; (4) study design as randomized controlled trial (RCT) or non-RCT; (5) study reporting at least one of the outcomes of interest, including the primary outcome i-PTH levels, and the secondary outcomes serum total calcium levels, ionized calcium amounts, phosphate concentrations, and peritonitis episodes, at 1- to 2-years of follow-up. Exclusion criteria were: (1) self-controlled study of concentration conversion between 1.25 mmol/L and 1.75 mmol/L dialysate calcium; (2) interventions combined with other treatments; (3) study without follow-up or with follow-up time below 12 months; (4) study without available statistical data.

### Study identification

First, all studies retrieved from the three databases were imported into EndNote version 7.0 (Thomson Reuters, New York, NY), with duplicates removed by automatic and manual deletions. Then, all titles of records after duplicate removal were viewed by two authors independently to exclude reviews/meta-analyses and obviously unrelated articles. Finally, full-text articles were reviewed to remove articles not conforming to the set eligibility criteria. A third investigator was involved in case of discrepancy.

### Data extraction and quality assessment

The following data were extracted independently by two authors from each study: first author’s name, year of publication, study design, PD pattern, concentration of dialysate calcium, sample size, mean patient age at study entry, follow-up time, dropouts, and interested outcomes at baseline and 1- to 2-year follow-up. The methodological quality assessment of the included studies (both RCTs and non-RCTs) was carried out with the risk of bias tool of the Review Manager software (version 5.3, Nordic Cochrane Centre, Denmark) [15]. A third investigator was involved in case of discrepancy.

### Statistical analysis

All statistical analyses were conducted with the Stata version 12.0 software (Stata Corp., College Station, TX, USA). Subgroup analysis was based on study design (RCT and non-RCT). As the outcomes of interest were reported in different units, standardized mean differences (SMDs) with 95% confidence intervals (CIs) were used to describe the mean differences for continuous variables. Dichotomous outcomes were assessed using odds ratios (ORs) with 95% CIs. *P* < 0.05 was considered statistically significant. Potential heterogeneity among studies was examined by Cochran’s Q [16] and *I*^2^ statistics [17]. A *P* value for heterogeneity < 0.10 or *I*^2^ > 50% indicated statistically significant heterogeneity. The random-effects model was then used for analysis. Sensitivity analysis was performed to evaluate the stability of results obtained in the meta-analysis for each outcome. A Galbraith plot was used to determine the possible source of heterogeneity [18]. Publication bias was assessed by Egger’s [19] and Begg’s [20] tests, with significant publication bias reflected by *P* < 0.10. The “trim-and-fill” method was used to assess the results in case of publication bias.

## Results

### Literature search

**Fig. 1:**
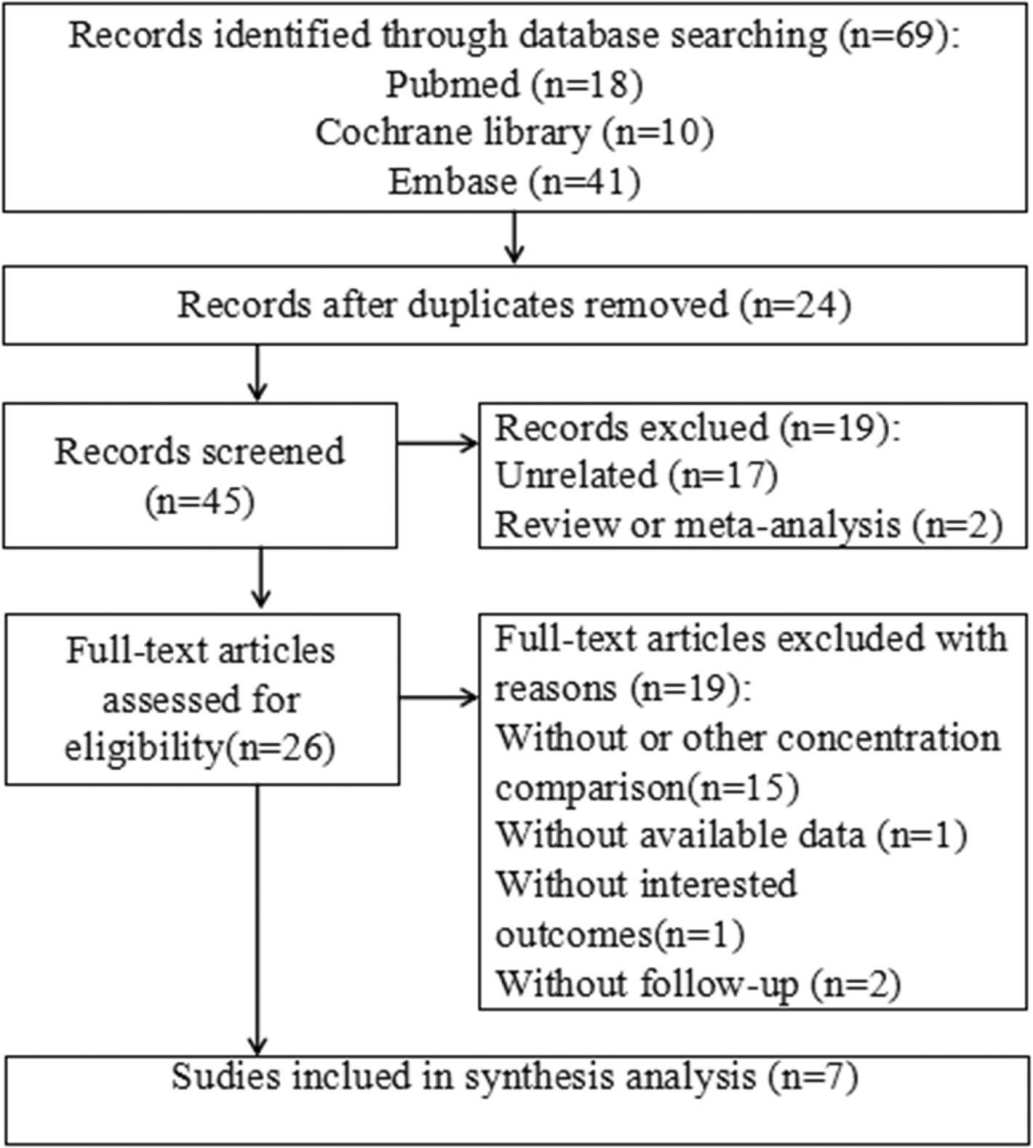
Flow diagram of the study selection process

A total of 69 studies (PubMed, 18; EmBase, 41, Cochrane Library, 10) were searched according to eligibility criteria. Forty-five hits remained for further screening after excluding duplicate studies. Then, 19 records were obviously unrelated to the topic (*n* = 17) or review/meta-analysis (*n* = 2), and excluded. Thus, 26 reports remained for full-text screening. Nineteen of them were excluded after full-text assessment for the following reasons: no comparison with other concentrations (*n* = 15), no available data (*n* = 1), no outcomes of interest (*n* = 1), and no or short (< 12 months) follow-up (*n* = 2). Finally, seven studies [11–13, 21–24] were included in the current meta-analysis. Four studies [11, 21–23] were RCTs, while three [12, 13, 24] were non-RCTs (Fig. 1).

### Study characteristics

Participants in six studies were CAPD patients, while one trial included PD patients. All studies involved comparisons between 1.25 mmol/L and 1.75 mmol/L dialysate calcium for PD patients. Six of the seven studies [11–13, 21–23] had a 12-month follow-up, and one [24] had a 24-month follow-up. Mean ages and dropout numbers showed no significant differences in various studies between the two groups. All the subjects in this meta-analysis had blood tests after fasting. The detailed study characteristics are listed in Table 1. Values at baseline and 1- to 2-year follow-up for the outcomes of interest are shown in Table 2. Three studies [11, 22, 24] provided the outcomes of interest in figures, which were imported into the Engauge Digitizer software (version 4.1) to convert data into mean ± standard deviation (M ± SD). The data were presented as M ± SD in one study [11], and mean ± standard error (M ± SE) in two studies [22, 24] after figure import into the software. Then, SDs were calculated based on sample size, mean, and SE. Meanwhile, one study [11] provided i-PTH levels for all patients. M ± SD was calculated using the Stata (version 12.0) software.

**Table 1:** Demographic characteristics of the included studies (1.25 group/1.75 group)

Study (year)	Study design	Nation	Participation	Sex ratio (M/F)	Intervention (mmol/L)	Sample size	Mean age (year)	Follow-up time (month)	Dropouts
Stein (1995) [21]	RCT	Britain	CAPD	29.l4/28.l 5	1.25/1.75	43/43	55.3 ± 2.1/ 54.2 ± 2.9	12	16/15
Johnson (1996) [22]	RCT	Australia	CAPD	5.6/3.8	1.25/1.75	22/23	59.8 ± 3.1/ 60.2 ± 2.8	12	11/12
Sa’nchez (2004) [23]	RCT	Spain	CAPD	NR	1.25/1.75	22/22	56 ± 11 (total)	12	8/12
Jing (2009) [11]	RCT	China	CAPD	13.20/8.16	1.25/1.75	33/24	58.6 ± 13.4/ 52.6 ± 15.6	12	2/2
Kang (2012) [24]	Non- RCT	South Korea	PD	22.23/104.86	1.25/1.75	46/190	49.2 ± 10.8/ 49.5 ± 13.2	24	0/0
Liang (2014) [12]	Non- RCT	China	CAPD	12.8/13.7	1.25/1.75	20/20	52.87 ± 12.0/ 57.0 ± 13.0	12	0/0
Wang (2016) [13]	Non- RCT	China	CAPD	13.17/14.16	1.25/1.75	30/30	56.75 ± 10.21/ 54.15 ± 7.75	12	0/0

*CAPD* Continuous ambulatory peritoneal dialysis, *F* female, *M* male, *NR* not report, *PD* peritoneal dialysis, *RCT* randomized controlled trial

**Table 2:** Comparisons of outcomes of interest at baseline and 1–2 year follow-up (1.25 group/ 1.75 group)

Study (year)	I-PTH		Total calcium		Ionized calcium		Phosphate		PE
	Baseline	Follow-up	Baseline	Follow-up	Baseline	Follow-up	Baseline	Follow-up	
Stein (1995)	NR	NR	NR	NR	NR	NR	NR	NR	17/43 vs 17/43
Johnson (1996)	33 ± 26.53/19 ± 23.22	35 ± 36.48/19 ± 16.58	2.63 ± 0.1/2.59 ± 0.2	2.50 ± 0.03/2.54 ± 0.23	1.26 ± 0.07/1.24 ± 0.07	1.30 ± 0.27/1.27 ± 0.13	1.57 ± 0.6/1.57 ± 0.56	1.84 ± 0.43/1.61 ± 0.3	12/22 vs 11/23
Sanchez (2004)	226.2 ± 228/98 ± 90.33	332 ± 188.67/86.7 ± 46.83	9.61 ± 0.92/9.73 ± 0.37	9.92 ± 1.04/10.04 ± 0.49	1.24 ± 0.08/1.25 ± 0.03	1.27 ± 0.03/1.29 ± 0.03	5.65 ± 1.3/5.61 ± 0.88	6.32 ± 1.47/5.49 ± 0.79	NR
Jing (2009)	124.5 ± 16.7/116.1 ± 14.3	191.86 ± 114.71/122.1 ± 104.4	2.78 ± 0.43/2.81 ± 0.51	2.25 ± 1.15/2.75 ± 1.52	NR	NR	2.14 ± 0.35/2.08 ± 0.26	1.61 ± 1.26/1.99 ± 1.71	1/33 vs 1/24
Kang (2012)	171.9 ± 250.8/166.6 ± 163.4	213.2 ± 147.7/145.2 ± 155.4	2.14 ± 0.16/2.16 ± 0.22	2.12 ± 0.15/2.22 ± 0.13	0.76 ± 0.12/0.78 ± 0.14	0.73 ± 0.06/0.78 ± 0.07	1.18 ± 0.34/1.22 ± 0.39	1.46 ± 0.25/1.44 ± 0.28	NR
Liang (2014)	31.70 ± 17.57/35.15 ± 18.67	30.88 ± 15.89/24.06 ± 15.21	7.78 ± 1.20/8.08 ± 1.01	8.84 ± 1.19/8.98 ± 0.89	NR	NR	6.50 ± 1.32/6.08 ± 1.88	5.42 ± 0.88/5.74 ± 1.07	NR
Wang (2016)	33.21 ± 57.62/31.98 ± 52.27	36.68 ± 47.27/33.70 ± 38.34	2.40 ± 0.25/2.28 ± 0.25	2.39 ± 0.26/2.51 ± 0.33	NR	NR	1.89 ± 0.22/1.85 ± 0.46	1.54 ± 0.39/1.68 ± 0.50	NR

*I-PTH* Intact parathyroid hormone, *NR* not report, *PE* peritonitis episodes

### Methodological quality assessment

**Fig. 2:**
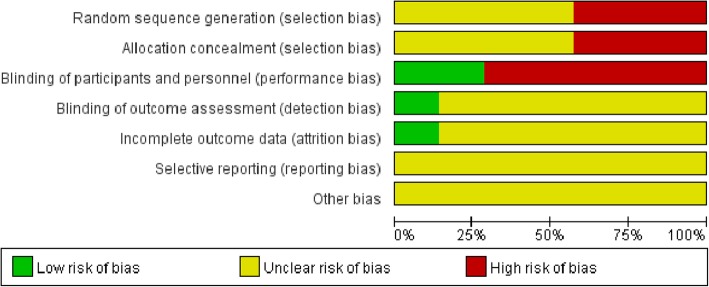
Risk of bias graph of included studies

**Fig. 3:**
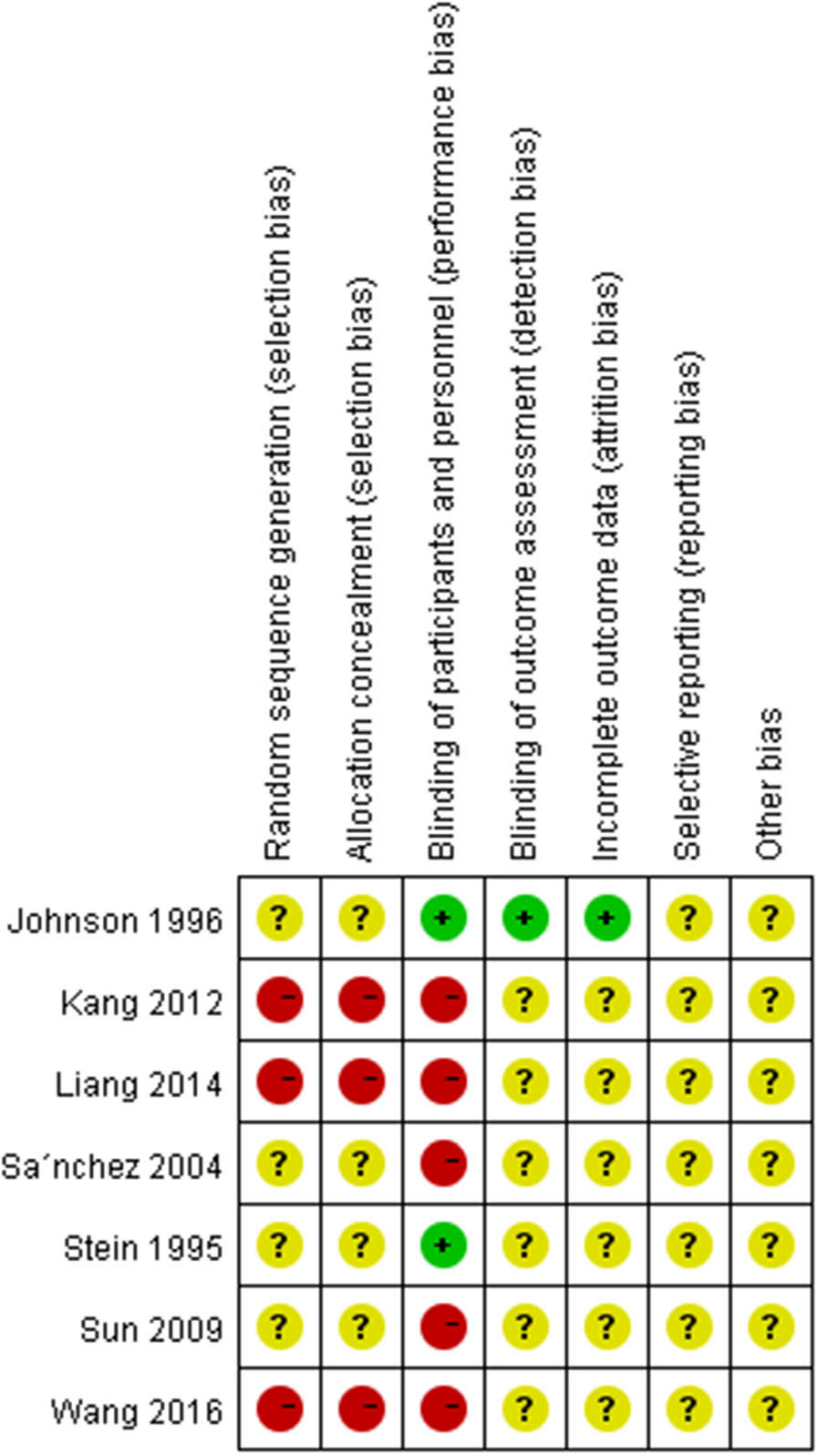
Risk of bias summary of included studies

The included studies underwent a quality assessment using the risk of bias tool of the Review Manager software (version 5.3, Nordic Cochrane Centre). An unclear and high risk of bias was found in random sequence generation and allocation concealment; meanwhile, a high risk of bias was obtained in blinding of participants, personnel, and outcome assessment, with an unclear risk of bias in incomplete outcome data and selective reporting, as well as other biases (Fig. 2). In particular, all four RCTs [11, 21–23] reported no specific method for random sequence generation and allocation concealment. Therefore, an unclear risk of selection bias existed in all four trials. In addition, all non-RCTs [12, 13, 24] used no method of sequence generation and allocation concealment. Hence, a high risk of selection bias was found in these studies. Two trials [21, 22] adopted blinding of participants and personnel, which resulted in a low risk of performance bias. The remaining five studies [11–13, 23, 24] did not use this method, and showed a high risk of performance bias. Only one trial [22] used blinding of outcome assessment and reported all outcomes of interest. Hence, unclear risk of detection and attrition biases were found in the remaining six studies. All studies showed unclear risk of reporting and other biases. Detailed results are shown in Fig. 3.

### Intact parathyroid hormone levels

**Fig. 4:**
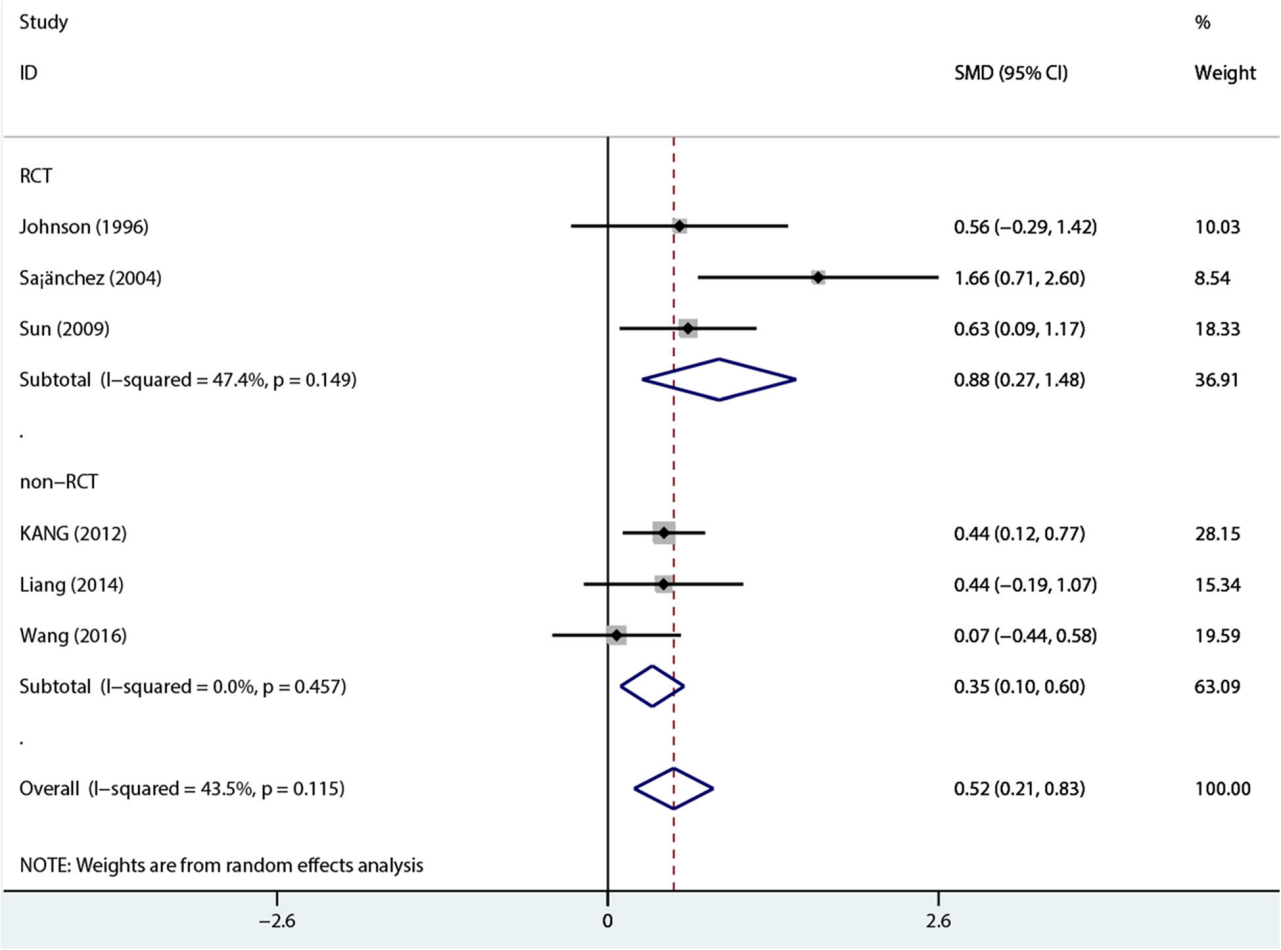
Comparison of i-PTH levels at 1- to 2-year follow-up between the two groups

A total of 439 patients (154 and 285 in the 1.25 and 1.75 mmol/L dialysate calcium groups, respectively) in 6 studies [11–13, 22–24] were included in pooled analysis. The results revealed that 1.75 mmol/L dialysate calcium significantly reduced i-PTH levels compared with the 1.25 mmol/L dose in PD patients (SMD = 0.519, 95%CI 0.207–0.831; *P* = 0.001) with low heterogeneity (*I*^2^ = 43.5%, *P* = 0.115). Subgroup analysis by study design showed a similar trend with the pooled analysis [SMD for RCTs, 0.88; 95%CI 0.27–1.48; *P* = 0.005 with low heterogeneity (*I*^2^ = 47.4%, *P* = 0.149); SMD for non-RCT, 0.35; 95%CI 0.1–0.6]; *P* = 0.006 with no heterogeneity (*I*^2^ = 0%, *P* = 0.457)] (Fig. 4). The random-effects model, which assumes that the true underlying effect varies among the included studies, was used for analysis.

### Total calcium levels

**Fig. 5:**
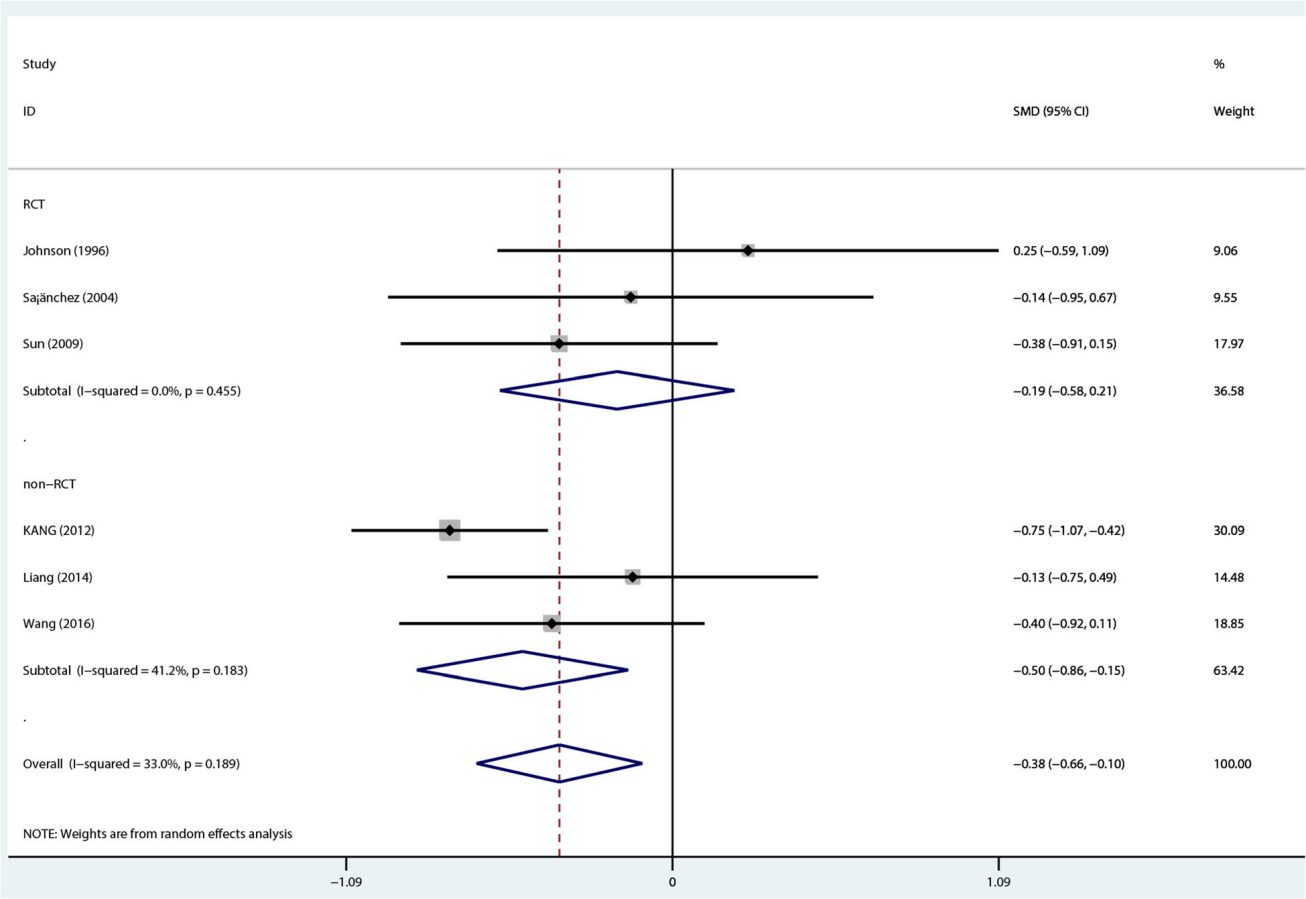
Comparison of total calcium levels at 1- to 2-year follow-up between the two groups

A total of 439 patients (154 and 285 in the 1.25 mmol/L and 1.75 mmol/L dialysate calcium groups, respectively) in 6 studies [11–13, 22–24] were included in pooled analysis. The summary results obtained by the random-effects model suggested that 1.25 mmol/L dialysate calcium was superior to the 1.75 mmol/L dose in decreasing serum total calcium levels in PD patients (overall SMD = − 0.378, 95% CI − 0.656 to − 0.101; *P* = 0.008) with low heterogeneity (*I*^2^ = 33.0%, *P* = 0.189). In non-RCT, SMD was − 0.5 (95%CI − 0.86 to − 0.15; *P* = 0.005) with low heterogeneity (*I*^2^ = 41.2%, *P* = 0.183). However, no statistically significant difference was found in RCTs (SMD = –0.19, 95% CI − 0.58 to 0.21; *P* = 0.355) with no heterogeneity (*I*^2^ = 0%, *P* = 0.455) (Fig. 5).

### Ionized calcium amounts

**Fig. 6:**
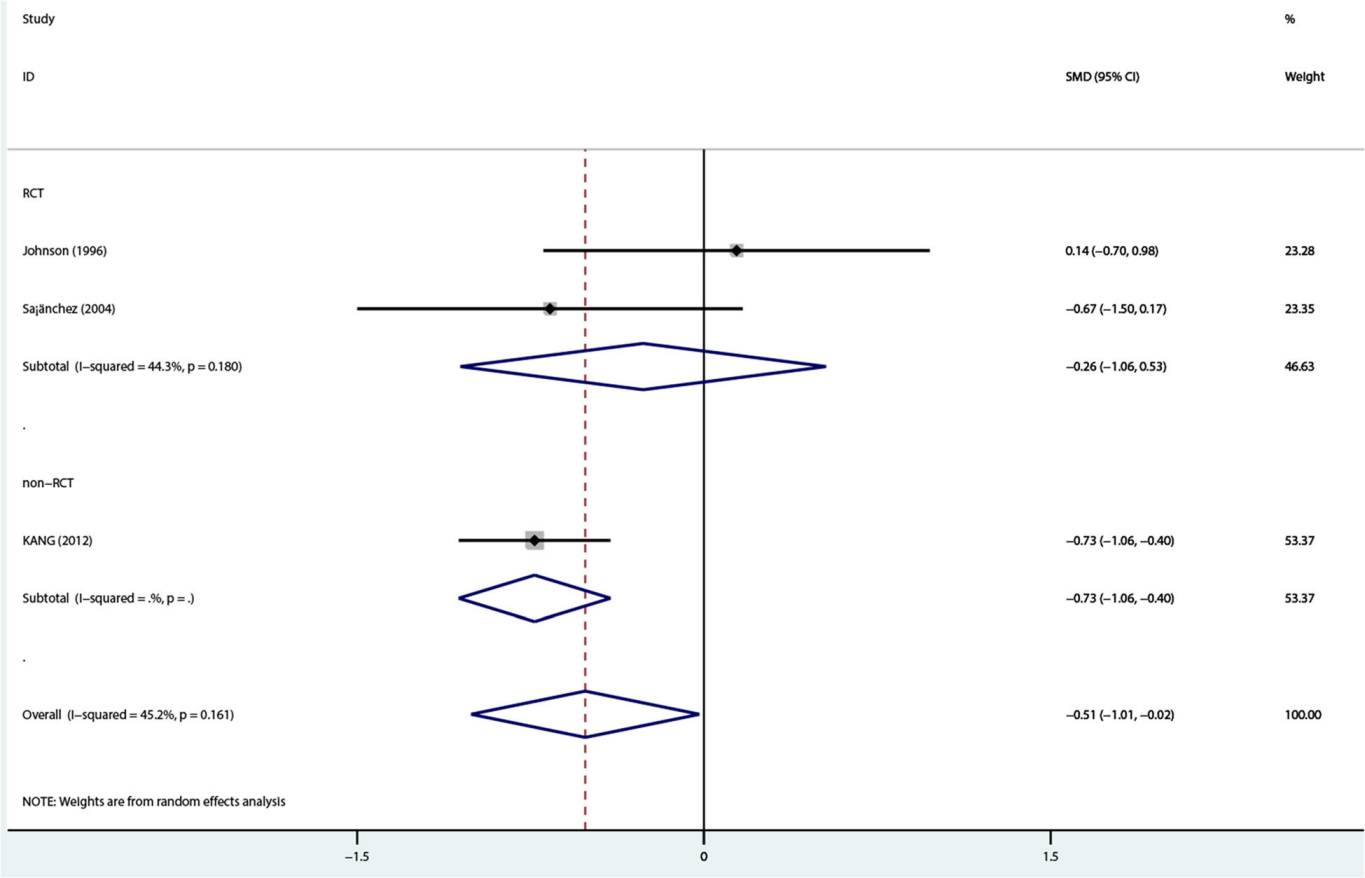
Comparison of ionized calcium levels at 1- to 2-year follow-up between the two groups

A total of 282 patients (71 and 211 in the 1.25 mmol/L and 1.75 mmol/L dialysate calcium groups, respectively) in 3 studies [22–24] were included in a synthesized analysis. The summary results by the random-effects model showed that 1.25 mmol/L dialysate calcium was superior to the 1.75 mmol/L dose in decreasing serum ionized calcium amounts in PD patients [overall SMD = –0.514, 95%CI − 1.009 to − 0.02; (*P* = 0.042), with low heterogeneity (*I*^2^ = 45.2%, *P* = 0.161). In non-RCT, SMD was − 0.73 (95%CI − 1.06 to − 0.4; (*P* = 0). However, no statistically significant difference was observed in RCTs (SMD = –0.26, 95%CI − 1.06 to 0.53; *P* = 0.515), with low heterogeneity (*I*^2^ = 44.3%, *P* = 0.18) (Fig. 6).

### Phosphate levels

**Fig. 7:**
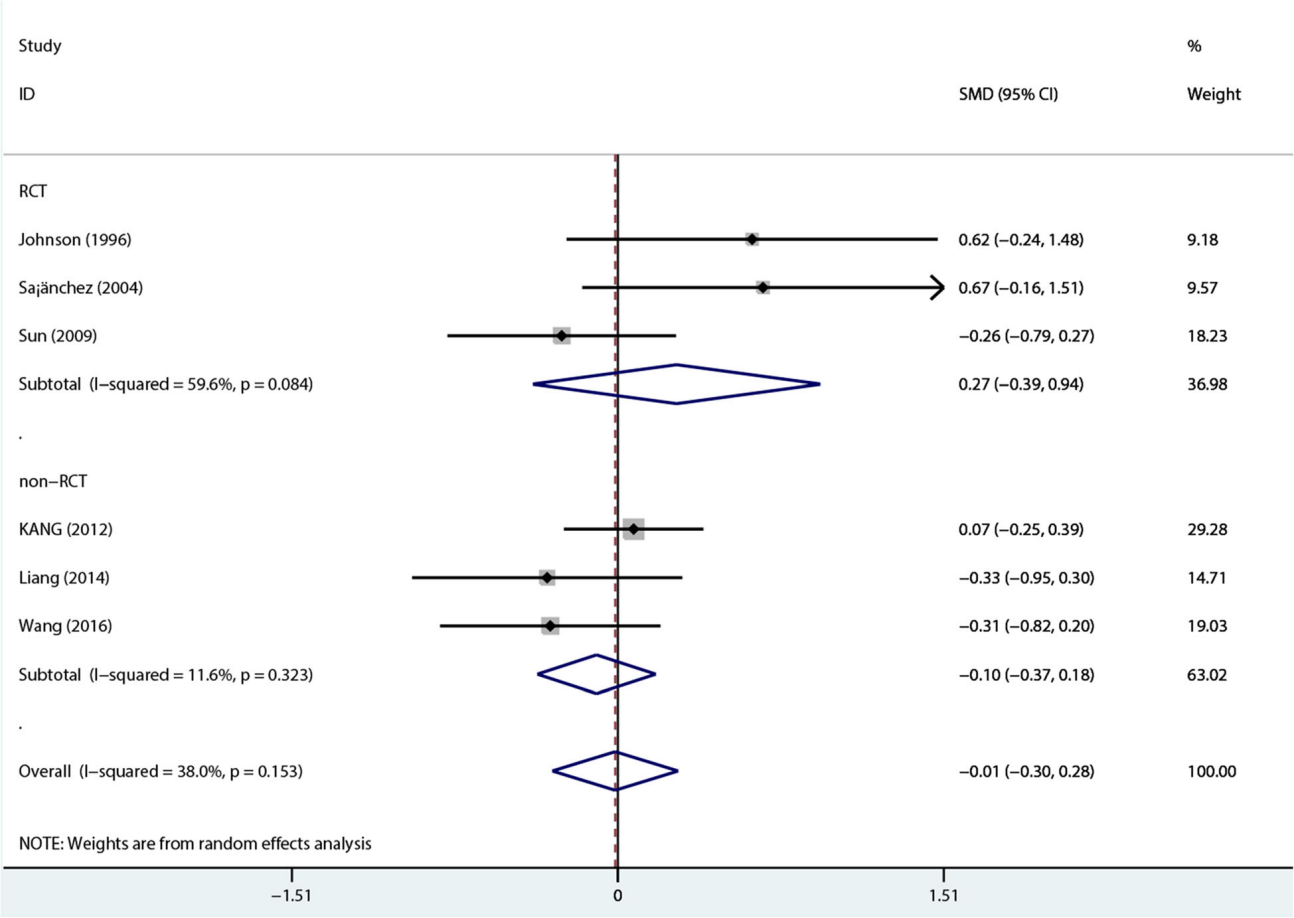
Comparison of phosphate levels at 1- to 2-year follow-up between the two groups

A total of 439 patients (154 and 285 in the 1.25 mmol/L and 1.75 mmol/L dialysate calcium groups, respectively) in 6 studies [11–13, 22–24]. The summary results by the random-effects model indicated no significant difference between the two groups in serum phosphate levels in PD patients [overall SMD = –0.012, 95%CI − 0.303 to 0.278; *P* = 0.934), with low heterogeneity (*I*^2^ = 38.0%, *P* = 0.153). In RCTs, SMD was 0.27 (95%CI − 0.39 to 0.94; *P* = 0.423), with medium heterogeneity (*I*^2^ = 59.6%, *P* = 0.084); in non-RCT, SMD was − 0.1 (95%CI − 0.37 to 0.18; *P* = 0.481), with low heterogeneity (*I*^2^ = 11.6%, *P* = 0.323) (Fig. 7).

### Peritonitis episodes

**Fig. 8:**
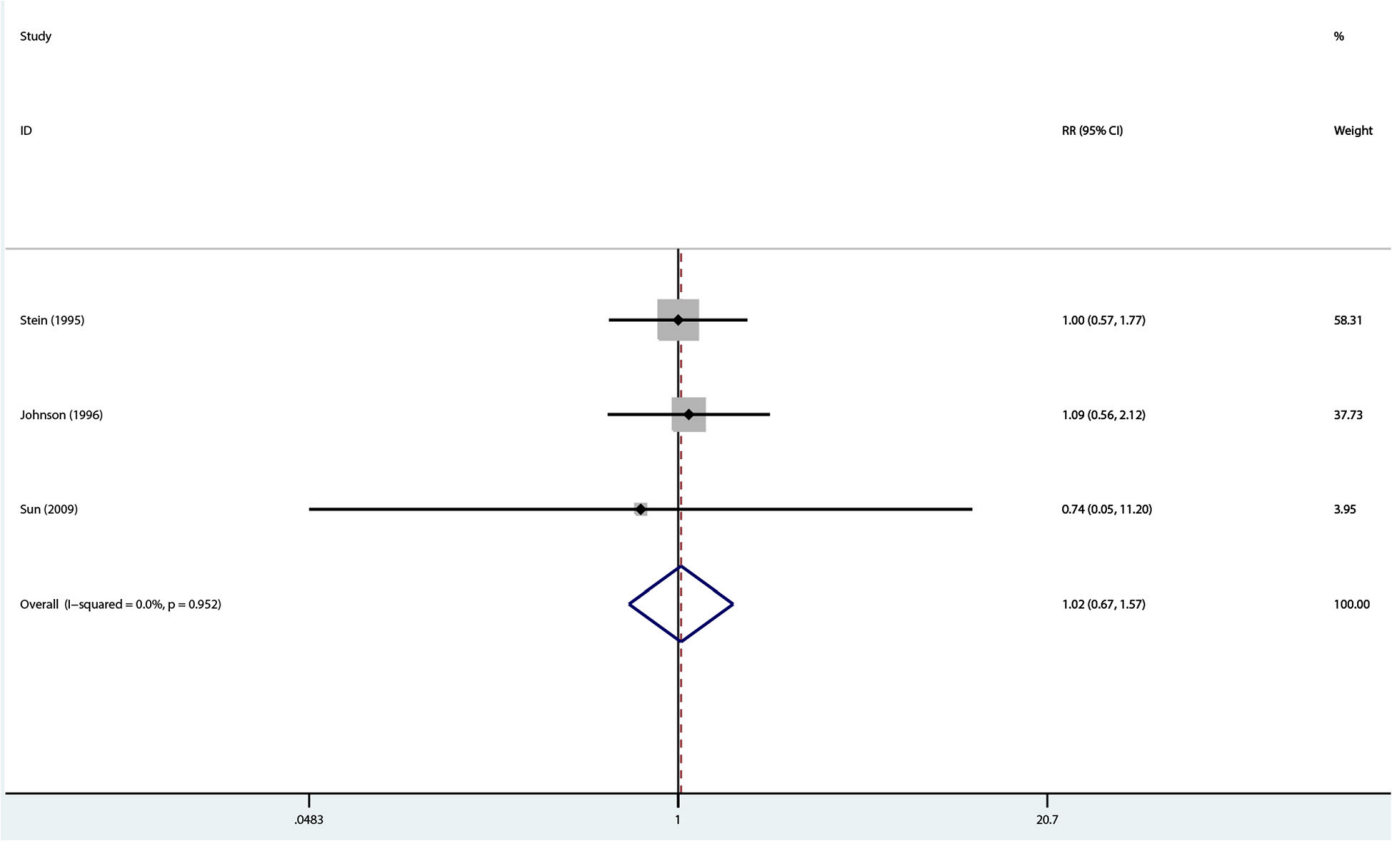
Comparison of peritonitis episodes at 1- to 2-year follow-up between the two groups

A synthesized analysis was possible in 188 of 439 patients, using the reference numbers of 21, 22, and 24. The 188 patients did not differ from those with unavailable data. The summary results by the random-effects model showed no significant difference between the two groups in peritonitis episodes in PD patients (OR = 1.034, 95%CI 0.563–1.9; *P* = 0.914), with no heterogeneity (I^2^ = 0%, *P* = 0.95) (Fig. 8).

### Sensitivity analysis

**Fig. 9:**
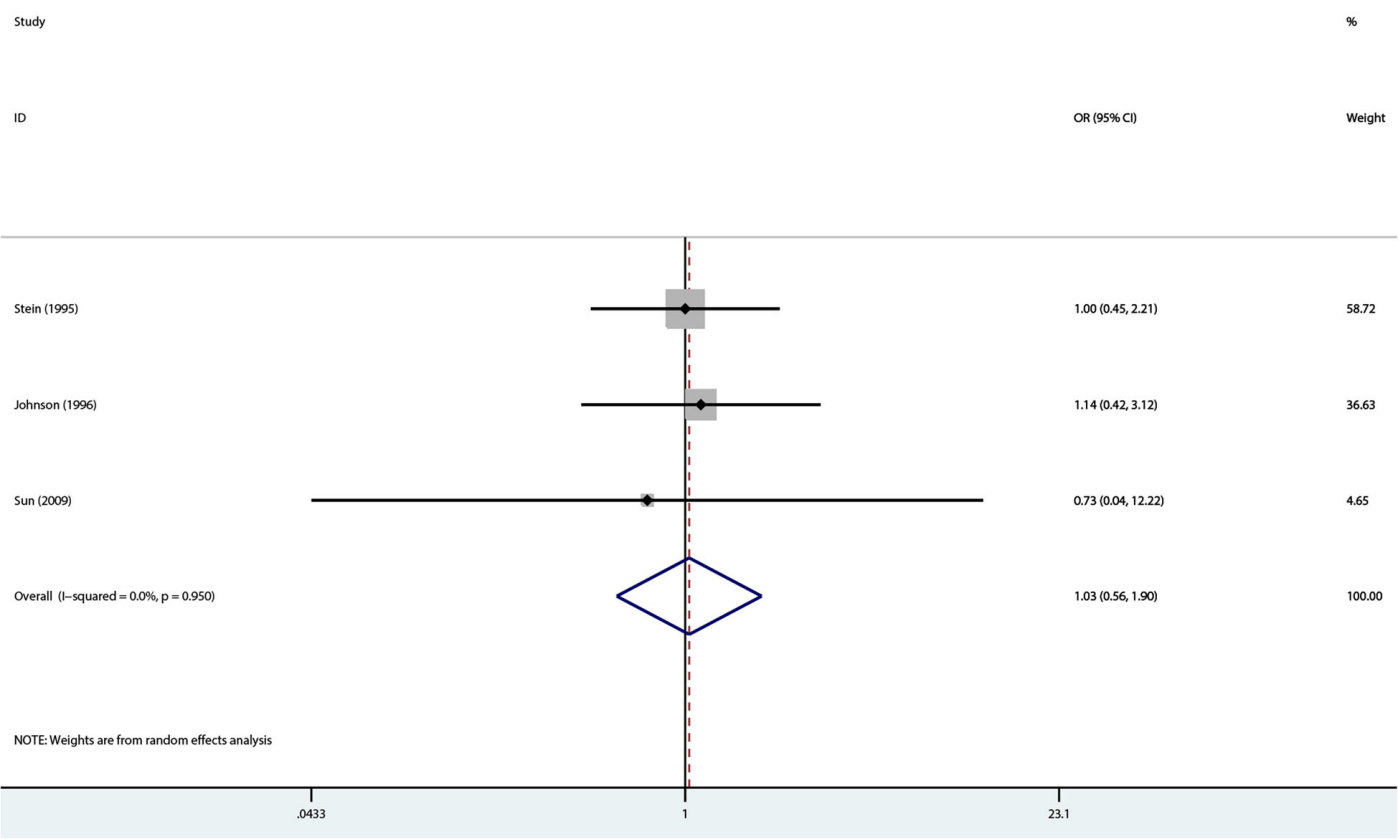
Galbraith plot for heterogeneity of total calcium levels at 1- to 2-year follow-up

**Fig. 10:**
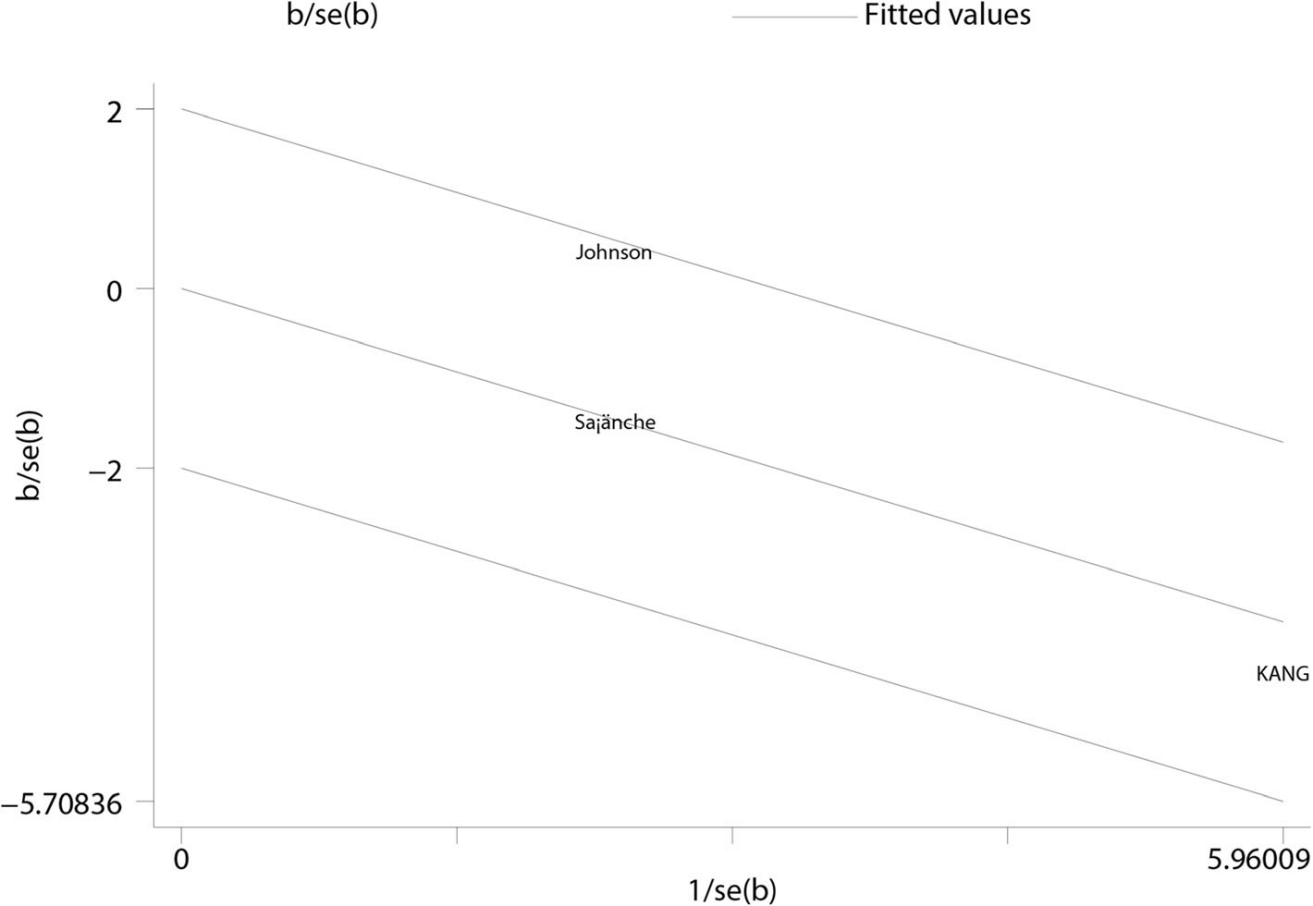
Galbraith plot for heterogeneity of ionized calcium levels at 1- to 2-year follow-up

**Fig. 11:**
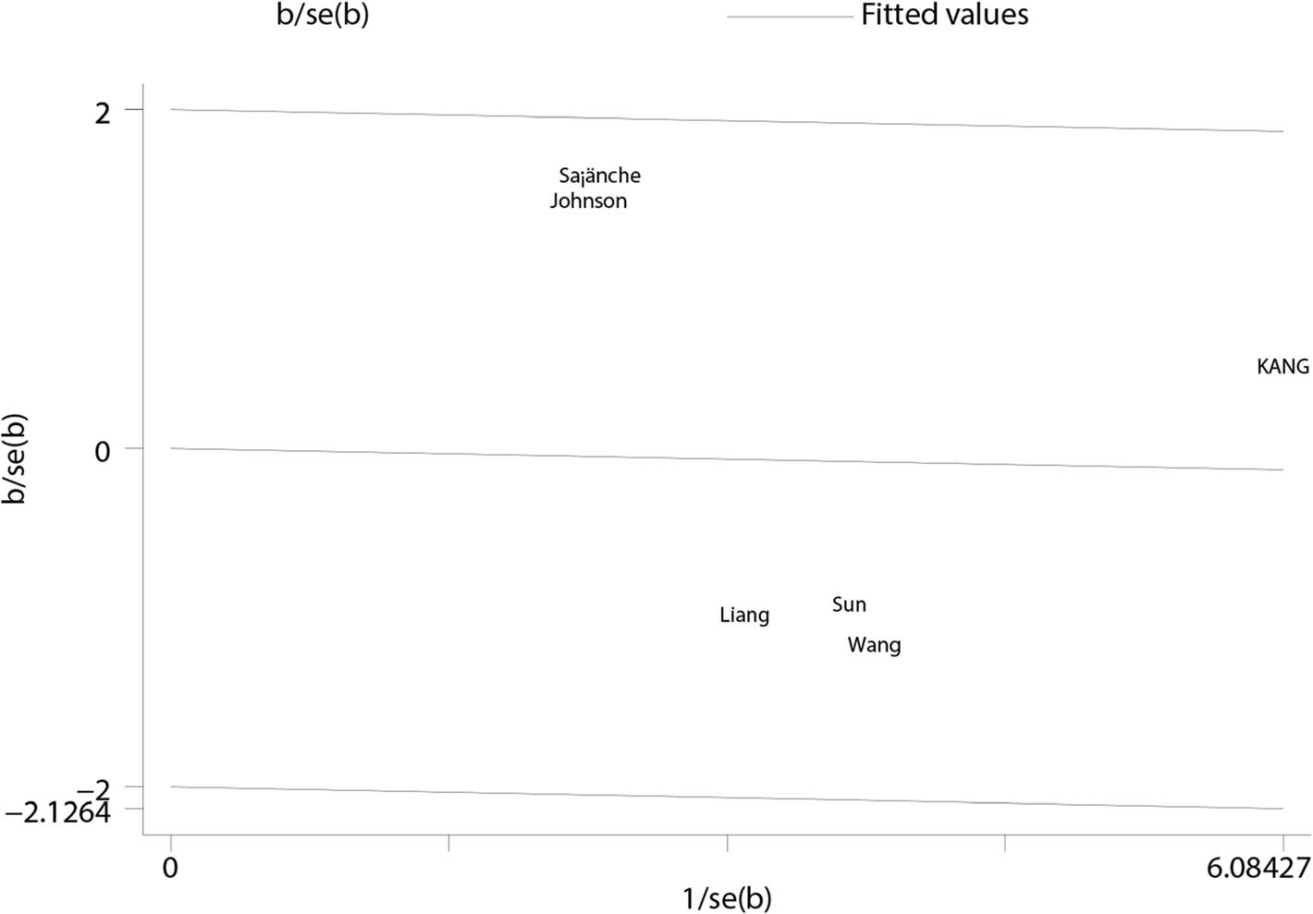
Galbraith plot for heterogeneity of phosphate levels at 1- to 2-year follow-up

**Fig. 12:**
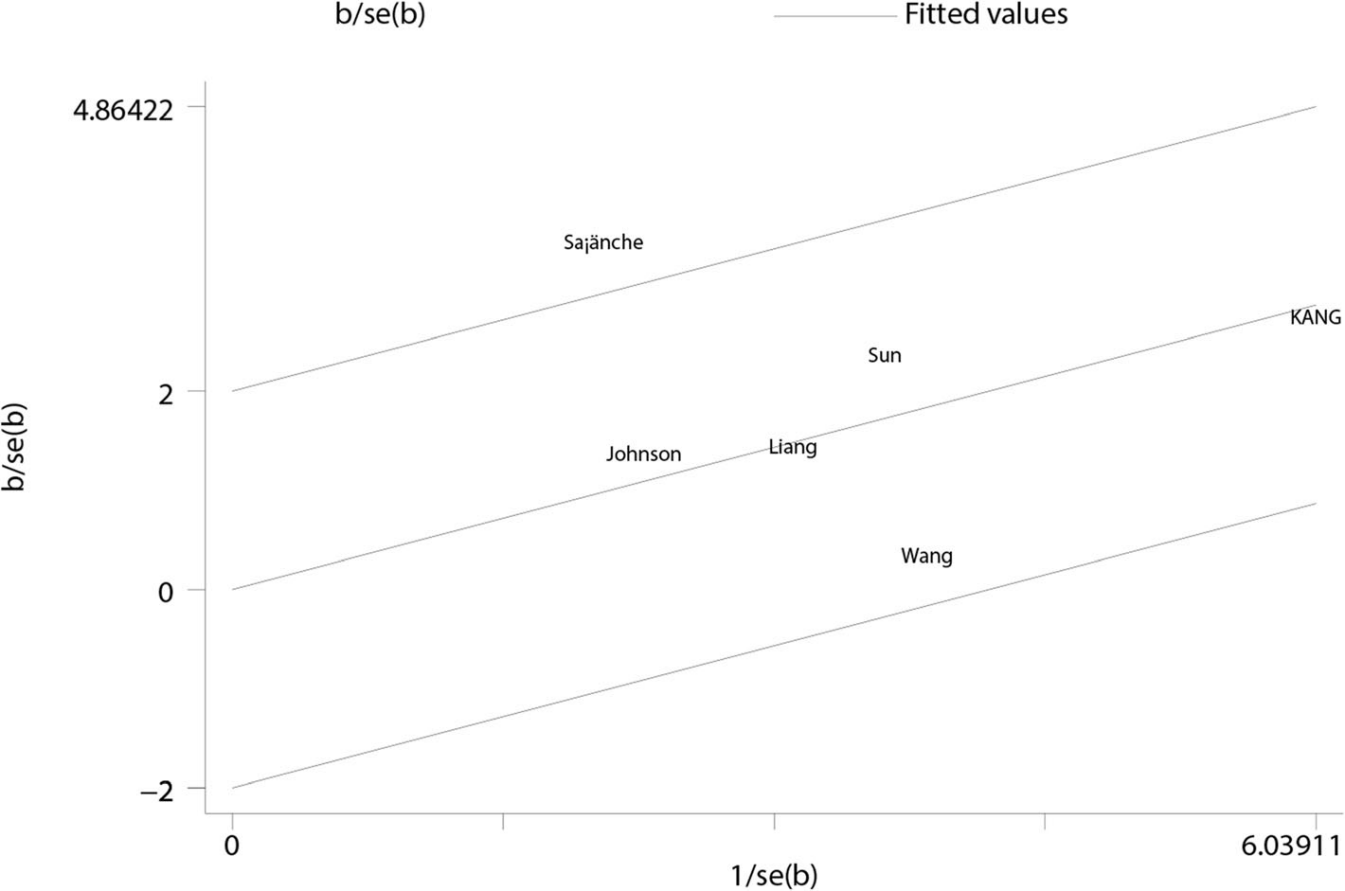
Galbraith plot for heterogeneity of i-PTH at 1- to 2-year follow-up

Sensitivity analysis was conducted for each outcome of interest, and conclusions were not affected by sequential exclusion of any specific study. No single study mainly conferred heterogeneity of total calcium (Fig. 9), ionized calcium (Fig. 10), and phosphate (Fig. 11) levels according to Galbraith plots. However, a study by Sanchez [23] was the main source of heterogeneity of i-PTH (Galbraith plot, Fig. 12). The full-text of the latter report was carefully assessed, and it was a multicenter study showing a significant difference in baseline i-PTH, which might have contributed to the observed heterogeneity.

### Publication bias

**Fig. 13:**
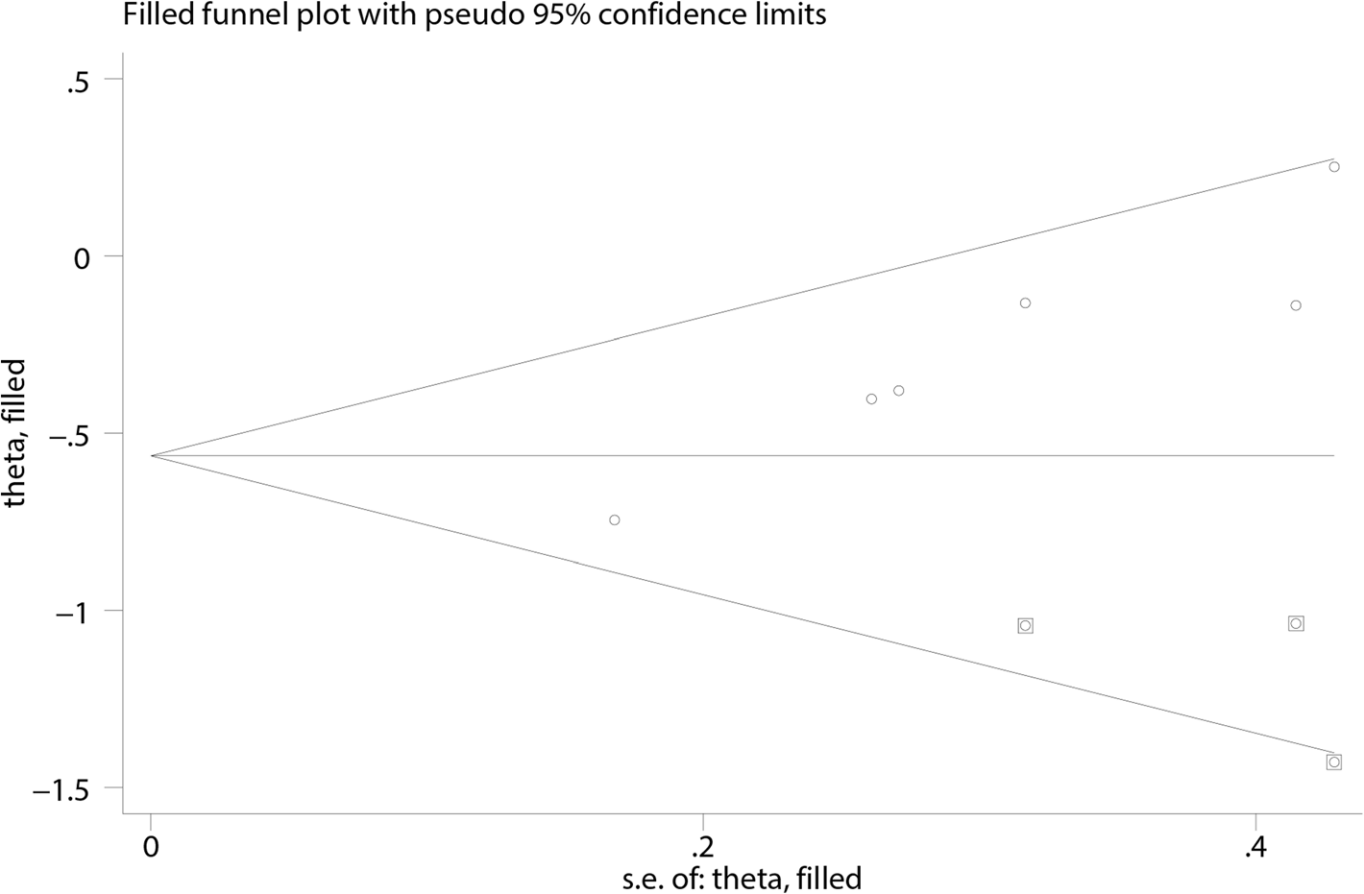
Filled funnel plot with pseudo 95% confidence limits of total calcium levels

Considering the small number of included studies, a potential publication bias was assessed by the Egger’s and Begg’s tests. The results suggested no evidence of publication bias for i-PTH levels, ionized calcium amounts, phosphate levels, and peritonitis episodes (Table 3). A significant publication bias was found for total calcium (*P* = 0.002 in the Egger’s test, and *P* = 0.024 in the Begg’s test). However, using the “trim-and-fill” method, the pooled outcomes were mathematically equivalent, although three studies were added for total calcium (in the fixed-effects model, *P* = 0 was obtained before and after; in the random-effects model, *P* = 0.008 and *P* = 0 were obtained before and after, respectively) [25]. After filling these three studies, the funnel plot became symmetrical, indicating the disappearance of publication bias (Fig. 13).

**Table 3:** Publication bias detected by the Egger’s and Begg’s tests

Test outcome	i-PTH	Total calcium	Ionized calcium	Phosphate	Peritonitis episodes
Egger’s test	*P* = 0.281	*P* = 0.002	*P* = 0.494	*P* = 0.571	*P* = 0.643
Begg’s test	*P* = 0.133	*P* = 0.024	*P* = 0.296	*P* = 0.452	*P* = 1.000

## Discussion

The main finding of this meta-analysis was that 1.75 mmol/L dialysate calcium could significantly reduce i-PTH levels compared with the 1.25 mmol/L dose in PD patients. However, 1.25 mmol/L dialysate calcium was superior to the 1.75 mmol/l dose in decreasing serum total calcium and ionized calcium amounts in PD patients. No significant differences in phosphate and peritonitis episodes were found between the two dialysate calcium concentrations.

A previous meta-analysis by Cao [10] and this study showed that low dialysate calcium is superior to high dialysate calcium in decreasing serum total calcium levels in PD patients, while no significant difference in phosphate amounts was found. This updated meta-analysis had new findings. First, four new studies [11–13, 21] were included in the previous meta-analysis, while the present study was more robust than the previous meta-analysis [10]. Secondly, all the studies included in this meta-analysis had comparisons between 1.25 mmol/L and 1.75 mmol/L dialysate calcium for PD patients, while the previous meta-analysis included one study comparing 1.0 mmol/L dialysate calcium with the 1.75 mmol/L dose for PD patients [26]. Thirdly, the summary results for i-PTH and peritonitis episodes were obtained, which was not the case in the previous meta-analysis. Finally, sensitivity, subgroup analyses, Galbraith plot, and Egger’s test were performed for each outcome to assess the stability of results, identify the main source of heterogeneity, and test publication bias, respectively.

The main function of PTH is to regulate the metabolism of calcium and phosphorus, which promotes blood calcium accumulation and decalcification of osteoclasts, while reducing blood phosphorus levels [27, 28]. i-PTH is the most common tool for monitoring the levels of PTH. The present study found that 1.75 mmol/L dialysate calcium significantly reduced i-PTH levels compared with the 1.25 mmol/L dose. Therefore, PD patients with secondary hyperparathyroidism were more indicated for 1.75 mmol/L dialysate calcium. Further, we found that 1.25 mmol/L dialysate calcium was superior to the 1.75 mmol/L dose in decreasing serum total calcium and ionized calcium levels in PD patients, although these findings might vary, according to sensitivity analysis. This could be explained by different patient characteristics, with or without ionized calcium measurements. Thirdly, PD patients with secondary hyperparathyroidism often have hypercalcemia. It is hard to decide which concentration of dialysate calcium is suitable for these patients, and further related studies are required. Fourthly, although no difference was found in phosphate amounts between the two dialysate calcium concentrations, hyperphosphatemia is common in ESRF patients receiving treatment for PD. Indeed, hyperphosphatemia is the main factor causing secondary hyperparathyroidism, and is strongly associated with serious cardiovascular complications such as coronary artery and heart valve calcification [29, 30]. Fifthly, the present study found no difference in peritonitis episodes between the two dialysate calcium concentrations, although this conclusion may be unreliable since small cohorts were included.

Although the overall- and non-RCT subgroup analysis findings for total and ionized calcium levels showed significant differences, RCT subgroup analysis for these two outcomes showed no statistical significance (Figs. 5 and 6). The two factors might have contributed to such results as follows: first, non-RCTs showed higher amounts compared with RCTs, especially a study by Kang [24] which contributed 30.09 and 53.37% to overall levels of total and ionized calcium, respectively. Secondly, one RCT [22] showed an opposite trend compared with the others. Therefore, more large-size randomized controlled trials (RCTs) are needed to verify the pooled results.

The main limitation of this study was the lack of large-sample RCTs. Selection and dropout biases existed in nonrandomized studies [12, 13, 24]. Meanwhile, all but one study [22] did not involve independent examiners, which might have contributed to observer bias and distortion (conscious or unconscious) in the perception or reporting of measurements [31]. Only two studies [21, 22] adopted blinding of participants and the personnel. Therefore, performance bias was found in the remaining five studies. In addition, ionized calcium, total calcium, phosphate, and intact PTH assays might affect the long-term effect of 1.25 versus 1.75 mmol/L dialysate calcium in PD patients, and these factors were not available in most included studies. Finally, background use of drugs might affect calcium-phosphorus metabolism. Such data were not available, and additional analysis was not conducted; this might alter the treatment effects between the two concentrations of dialysate calcium in PD patients.

## Conclusions

Overall, this study found 1.75 mmol/L dialysate calcium is superior in reducing PTH levels compared with the 1.25 mmol/L dose. Meanwhile, 1.25 mmol/L dialysate calcium was associated with better effects in PD patients with secondary hypercalcemia compared with the 1.75 mmol/L dose. Further well-designed and high-quality studies are required to determine suitable dialysate calcium concentration for patients with both hyperparathyroidism and hypercalcemia.

## Abbreviations

CAPD: Continuous ambulatory peritoneal dialysis
CIs: Confidence intervals
ESRF: End-stage renal failure
i-PTH: Intact parathyroid hormone
PD: Peritoneal dialysis
PTH: Parathyroid hormone
RCT: Randomized controlled trial
SMD: Standard mean difference
